# Clinical characteristics of Leucine-rich glioma-inactivated protein 1 antibody-mediated autoimmune encephalitis in a 6-year-old girl: case report and literature reviews

**DOI:** 10.1186/s12883-023-03299-z

**Published:** 2023-06-30

**Authors:** Liqing Chen, Tangfeng Su, Yan Liu

**Affiliations:** Department of Pediatrics, Tongji Hospital, Tongji Medical College, Huazhong University of Science and Technology, Wuhan, China

**Keywords:** LGI1, Autoimmune encephalitis, Seizures, Pediatric, Case report

## Abstract

**Background:**

Autoimmune encephalitis related to the leucine-rich glioma-inactivated protein 1(LGI1) antibody is the most prevalent in older adults, manifesting as seizures, faciobrachial dystonic seizures (FBDS), cognitive impairment, memory disturbance, hyponatremia and neuropsychiatric disorders. However the data pertaining to children affected by the disease is still limited.

**Case presentation and literature reviews:**

This study presents a detailed report of a 6-year-old Chinese girl who experienced nose aches and faciobrachial dystonic seizures (FBDS). Electrolyte testing revealed that she had hyponatremia and brain MRI showed an abnormality in the left temporal pole. Additionally, anti-LGI1 antibodies were detected in both her serum (1:100) and CSF (1:30). The patient was treated with immunotherapy and symptom management, which proved effective. Furthermore, we provide a summary of 25 pediatric cases of anti-LGI1 encephalitis. Pediatric patients rarely exhibited FBDS and hyponatremia, and some cases presented with isolated syndromes. But the therapeutic outcomes of pediatric patients were generally good.

**Conclusions:**

In this report, we describe a patient who developed a rare symptom of nose aches possibly as one of symptoms of anti-LGI1 encephalitis, which highlights the possibility of atypical symptoms in children that may be misdiagnosed. Reviewing the literature, the clinical features differed between pediatric and adult cases. Therefore, it is crucial to collect and analyze data from more cases to promote accurate diagnosis and timely treatment.

## Background

The initial report on autoimmune encephalitis related to the LGI1 antibody was published by Lai et al in 2010 [1]. Anti-LGI1 encephalitis is considered the second most common form of autoimmune encephalitis observed in adults with an average onset age of around 63 years and a higher incidence in males [2, 3]. The main clinical features of anti-LGI1 encephalitis are seizures, particularly faciobrachial dystonic seizures (FBDS), cognitive impairment, memory disturbance, hyponatremia and neuropsychiatric disorders [4]. Reports on pediatric anti-LGI1 encephalitis are rare. Lopez-Chiriboga et al. examined neurological autoimmunity of LGI1 and contactin-associated protein like-2 (CASPR2) in 264 voltage gated potassium channel (VGKC) complex IgG seropositive pediatric patients and found that only 2% of patients tested positive for LGI-IgG [5]. The rarity of anti-LGI1 encephalitis in pediatric patients may be related to a lower expression of LGI1 protein in younger individuals as observed in mice [6]. We present the case of a 6-year-old Chinese girl who was diagnosed with anti-LGI1 encephalitis which was characterized by seizures and hyponatremia. Additionally, we conducted an analysis to summarize the clinical features of previously reported cases of pediatric anti-LGI1 encephalitis.

## Case presentation

**Fig. 1:**
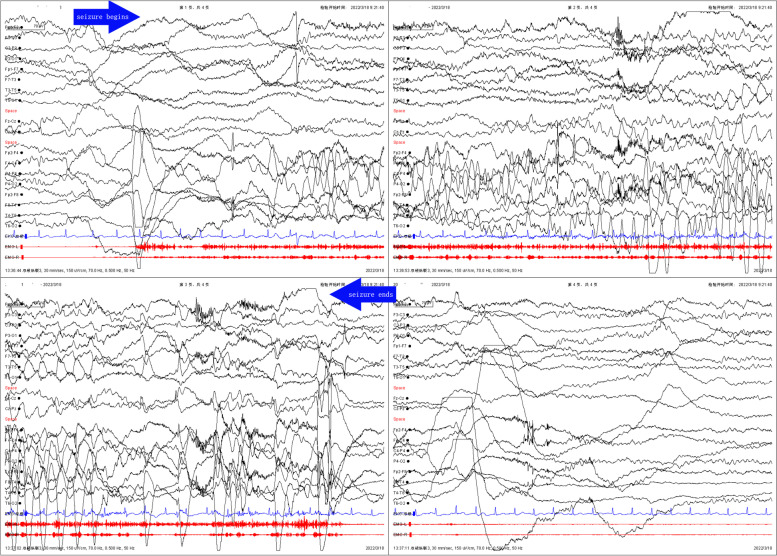
Video electroencephalogram recordings before the treatment: fast wave rhythm→ slow wave rhythm at the left frontal-temporal electrodes, evolved to the left hemisphere. The whole procedure lasted for 20-25 seconds. Moreover, a tonic twitch of the right upper arm, associated with scared facial expression and frequent head shaking were observed clinically (lasting for 20-25 seconds)

**Fig. 2:**
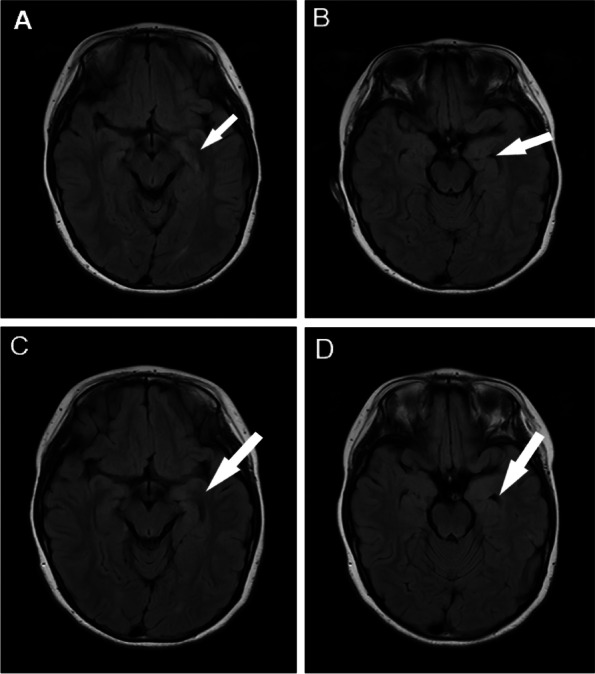
The results of brain MRI. **A**, **B** Brain MRI showed increased signal in the left temporal pole before treatment. **C**, **D** Brain MRI showed increased signal in the left temporal pole after intravenous immunoglobulin and corticosteroids therapy. Picture **A** was compared with picture **C**, the high signal slightly decreased and the edema slightly subsided in the left temporal pole (the white arrow) on T2FLAIR. Picture **B** was compared with picture **D**, the temporal horn (the white arrow) was enlarged in the picture **D**. It indicated that edema subsided after treatment

**Fig. 3:**
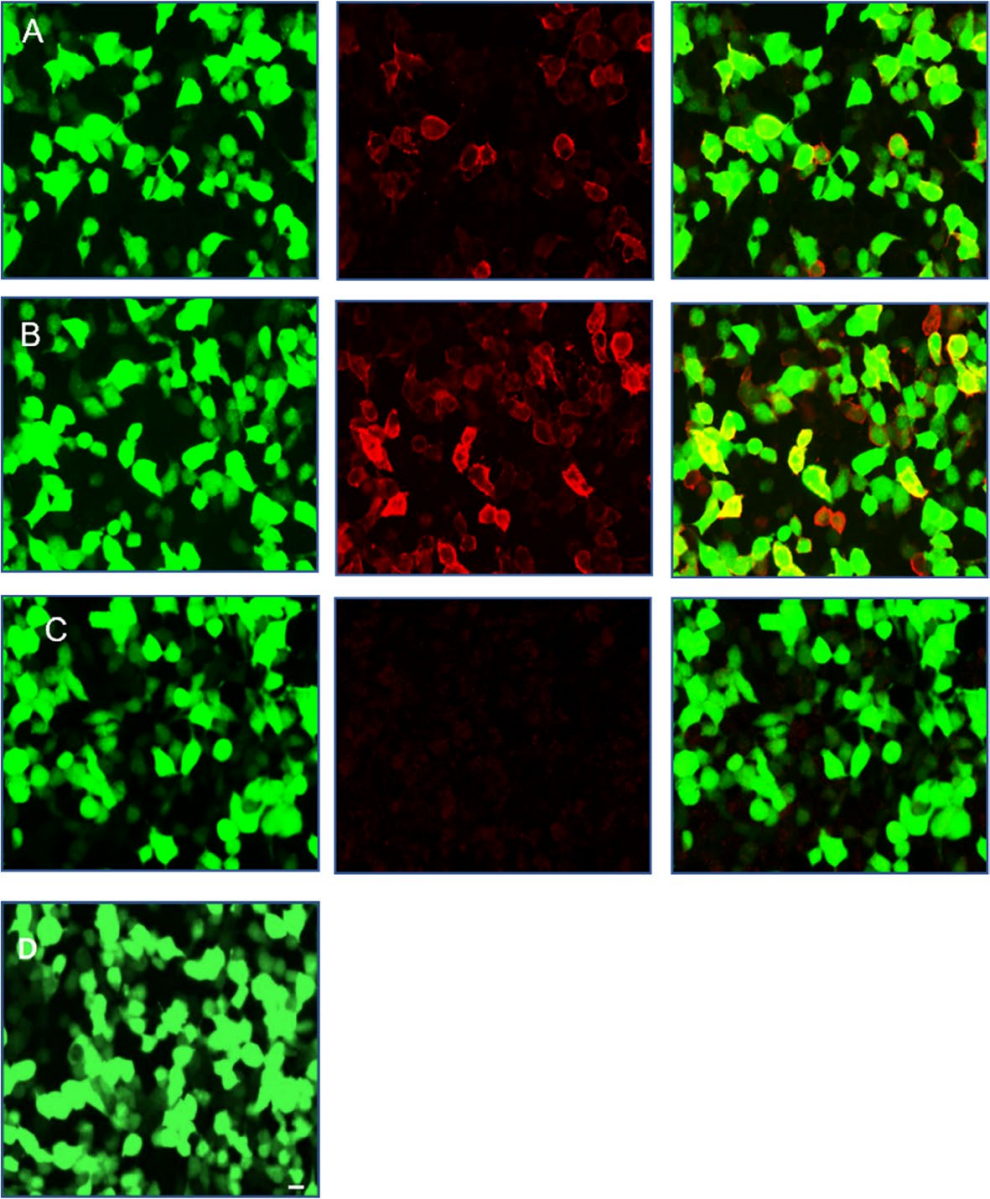
The levels of anti-LGI1 antibody were measured in both serum and CSF before treatment. **A** The cell-based assay for CSF anti-LGI1 antibody showed a positive result with a titer of 1:30. **B** The cell-based assay for serum anti-LGI1 antibody showed a positive result with a titer of 1:100. **C** Negative control. **D** Scale bar: 20um

Our hospital’s emergency department admitted a previously healthy 6-year-old Chinese girl who was experiencing recurrent seizures. The patient had no family history of seizures or any other neurological disorders. The patient began experiencing intermittent nose aches one month ago. She experienced nose aches once every 2-3 days, which subsided after approximately 10 seconds each time. Initially, she received a diagnosis of rhinitis and was prescribed mometasone furoate at another hospital. This treatment appeared to initially reduce the frequency of nose ache, but it worsened again half a month ago, occurring as frequently as 1-2 times per day. A week ago, she also developed a cough and was promptly given oral anti-cough and anti-inflammatory medication, which led to significant improvement. A few hours prior to admission, the patient experienced frequent seizures characterized by a tonic twitch of her right upper arm, accompanied by a frightened facial expression and frequent head shaking. Each seizure episode lasted only a few seconds. The patient experienced seizures at a frequency of approximately 10-20 times per day, but did not lose consciousness during these episodes. The patient did not present any psycho-behavioral changes, sleep disorders, cognitive impairment, or other related symptoms. Our neurological examination did not reveal any positive signs or obvious abnormalities. After conducting a video electroencephalogram (VEEG) that showed epileptiform activity in the left frontotemporal region (Fig. 1), Perampanel was prescribed at a dosage of 2mg per day. This led to a reduction in her seizures about two days following the commencement of the treatment. However, she continued to experience nose aches once or twice per day. A brain magnetic resonance imaging (MRI) was conducted on the patient, and the result revealed an increased signal in the left temporal pole on T2FLAIR (Fig. 2 AB). The laboratory examinations, including a complete blood count, liver and renal function tests and electrolyte and metabolic tests, showed normal results. The initial serum sodium level was measured at 138.2mmo/L. The cell count, glucose, protein and chloride levels in the cerebrospinal fluid (CSF) sample were all within normal limits. The initial sodium level in CSF was 146.7mmol/L and no pathogens growth was observed in the sample. The cell-based indirect immunofluorescence antibody assay was utilized to evaluate the presence of antibodies for autoimmune encephalitis in the serum and CSF samples. This approach revealed the existence of Anti-LGI1 antibodies in both the serum (1:100) and CSF (1:30) (Fig. 3 AB). Other series of autoimmune encephalitis antibody were detected to be negative in serum and CSF. In addition, tests for tumor maker and paraneoplastic neuronal antibody showed a negative result. Computed Tomography (CT) scans of the chest and thymus also showed a negative result.

**Fig. 4:**
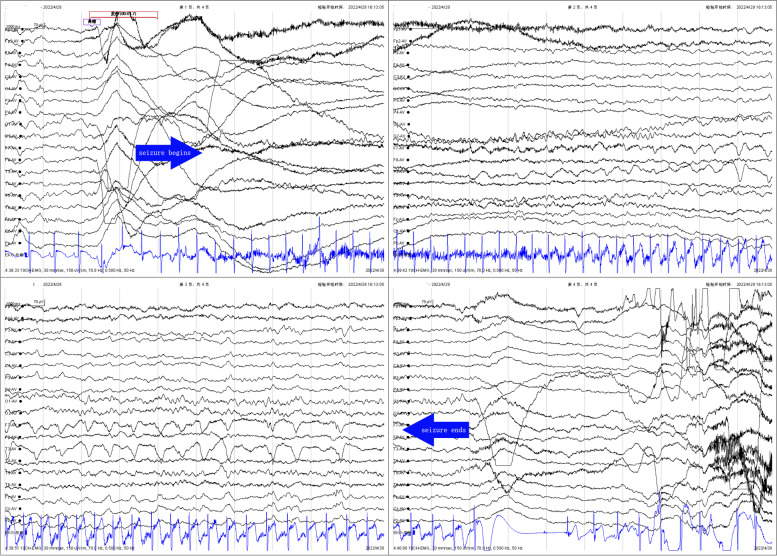
Video electroencephalogram recordings after one month of treatment: slow wave and sharp slow wave in left temporal region, lasting for 20 seconds. At the same time, the patient complained of nasal pain, lasting for about 20 seconds

**Fig. 5:**
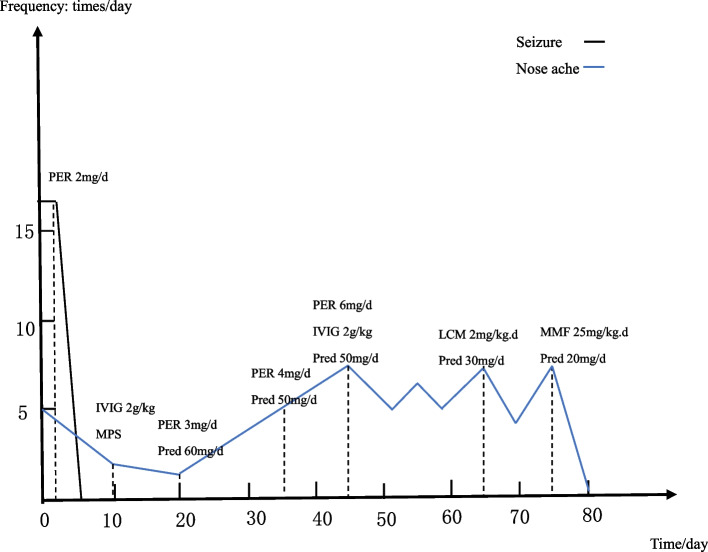
A timeline figure of symptoms and treatment. The black line on the graph represents the frequency of a tonic twitch of the patient’s right upper arm. The blue line on the graph represents the frequency of nose aches. The dotted line on the graph represents the timeline of medication usage. MPS is used as 20mg/kg.d for 3 days, 10mg/kg.d for 3 days, 5mg/kg.d for 3 days. PER: Perampanel; IVIG: Intravenous Immune Globulin; MPS: Methylprednisolone; Pred: Prednisone; LCM: Lacpsamide; MMF: Mycophenolate Mofetil

The patient found immediate relief from the tonic twitch in her right upper arm after taking 2mg/day of Perampanel. However, she continued to experience nose aches 2-7 times a day. The dosage of Perampanel was gradually increased up to 6mg/day. The patient also received intravenous immunoglobulin (IVIG, 2g/kg) and methylprednisolone pulse therapy (20mg/kg.d for 3 days, 10mg/kg.d for 3 days, 5mg/kg.d for 3 days). The patient was subsequently prescribed oral prednisolone, starting at 2mg/kg.d for 2 weeks and then reducing 2 tablets every 2 weeks. Unfortunately, this treatment did not yield any significant benefits. After one month of undergoing a treatment regimen consisting of Perampanel, IVIG and corticosteroids, the patient’s VEEG, brain MRI and anti-LGI1 antibodies were re-evaluated. The results of the VEEG (Fig. 4) revealed a slow wave in the left temporal region, accompanied by a sharp slow wave during the onset of nose ache. The brain MRI showed a minor reduction in edema in the left temporal pole (Fig. 2 CD). The titers of the anti-LGI1 antibody decreased in both the serum (1:30) and the CSF (1:10). Afterward, the patient received another IVIG (2g/kg) infusion and lacosamide (2mg/kg.d) was added to the treatment regime. Although the frequency of nose aches decreased slightly (2-3 times/day), the patient started experiencing dizziness and headaches. Unfortunately, after a week, the frequency of nose aches increased again and was observed to occur between 3-7 times per day. Throughout the patient’s treatment, regular complete blood count, liver and renal function, and electrolyte tests were conducted. The serum sodium level was found to be 133mmol/L. We escalated therapy with mycophenolate mofetil (MMF, 25mg/kg.d). As a result, her nose ache disappeared (Fig. 5). The patient continues to be closely monitored and has not experienced a recurrence of her symptoms.

### Literature review of pediatric patients with anti-LGI1 encephalitis

A literature search was conducted on Pubmed, CNKI, WOS and WanFang (until June 2023), using the search terms ‘children or pediatric’ and ‘leucine-rich glioma inactivated 1 or LGI1’. Following a careful review of the literature, we identified 15 articles describing 24 pediatric patients (<18 years) with anti-LGI1 encephalitis [5, 7–20]. Our patient was included as the 25th participant in the study.

The twenty-five cases described included 15 females and 10males. The median age of onset is 9.84, ranging from 2 to 17 years. The clinical characteristics of these pediatric patients with anti-LGI1 encephalitis are summarized in Tables 1 and 2. The prominent clinical manifestations include seizures, encephalopathy, psycho-behavioral changes, movement disorders. Only 4 patients had FBDS [15, 17, 20] and 3 patients had hyponatremia [15, 18] which are commonly encountered in adult LGI1-IgG-positive patients. While frequent seizures, encephalopathy and psycho-behavioral changes are common neurological accompaniments of anti-LGI1 encephalitis in pediatric patients, it is worth noting that some patients just present with isolated symptoms, such as movement disorders, sleep disorders, seizures, or cerebellar ataxia.

**Table 1:** The clinical characteristics of 25 patients

Pt/Sex/ Age of onset	Clinical features	EEG	Brain MRI/PET	Treatment		Outcome	Ref
1/M/6y	Movement disorders	Normal	Normal	Pimozide, tetrabenazine	CS, IVIG	Recovered	Erer Özbek S et al (2015) [7]
2/F/8y	Encephalopathy, seizures, psycho-behavioral changes	Epileptiform discharge	MRI: hyperintense lesions in the right hippocampal region	LEV	CS, IVIG	Recovered	Incecik F et al (2016) [9]
3/F/7y	Psycho-behavioral changes, encephalopathy, sleep disorders, movement disorders	Background slowing	Normal	-	CS, IVIG	Recovered	AlHakeem AS et al (2016) [8]
4/M/14y	Psycho-behavioral changes, amnesia, seizures	Normal	MRI: swelling of the left hippocampus	-	CS, PE, MMF	Improved	Schimmel M et al (2018) [10]
5/M/9y	Encephalopathy, seizures, dizzy, psycho-behavioral changes	Epileptiform discharge	MRI: Brainstem: T2 hyperintensity without enhancement	-	IVIG	Improved	López-Chiriboga AS et al (2018) [5]
6/F/17y	Encephalopathy, sleep disorders, pain, ANS	-	Normal	-	IVIG	Improved	López-Chiriboga AS et al (2018) [5]
7/F/17y	Encephalopathy, seizures	Epileptiform discharge	Normal	-	IVIG, Aza	Improved	López-Chiriboga AS et al (2018) [5]
8/F/5y	Encephalopathy, seizures, psycho-behavioral changes	Epileptiform discharge	Normal	VPA, LCM	-	Seizures cessation	López-Chiriboga AS et al (2018) [5]
9/M/13y	Encephalopathy, amnesia, seizures, pain, psycho-behavioral changes	Normal	MRI: Left limbic T2 hyperintensities	-	IVIG, CS	Improved	López-Chiriboga AS et al (2018) [5]
10/F/17y	Weight loss, pain, ANS	Normal	N/A	Symptomatic	-	N/A	López-Chiriboga AS et al (2018) [5]
11/F/14y	Dizzy, pain, psycho-behavioral changes	Normal	Normal	Symptomatic	-	Stable	López-Chiriboga AS et al (2018) [5]
12/F/7y	Seizures (including FBDS), encephalopathy, hyponatremia, amnesia	Epileptiform discharge	PET: subtle hypometabolism in the right temporal and bilateral parietal cortices	LEV, VPA, CBZ, midazolam, TP, PB, PHT, LCM; TPM	CS, IVIG, R-FC	Relapsed	Mir A et al (2019) [11] Alotaibi W (2022) [15]
13/M/8y	Sleep disorder	Focal slow waves	MRI: left hippocampal lesions	-	IVIG	Improved	Zhang J et al (2019) [12]
14/M/15y	Seizures	Focal slow waves and epileptic form discharge	Normal	LEV,	IVIG	Recovered	Zhang J et al (2019) [12]
15/F/14y	Sleep disorders, movement disorders, psycho-behavioral changes	N/A	Normal	N/A	N/A	N/A	Giannoccaro MP et al (2021) [13]
16/F/4y	Seizures, encephalopathy, sleep disorders, psycho-behavioral changes	epileptiform discharge	normal	LEV, CZP, OXC;	IVIG, CS	Recovered	Luo J et al (2021) [14]
17/F/7y	Seizures	Background slowing and epileptiform discharge	normal	OXC	IVIG, CS	Recovered	Wang Y et al (2022) [16]
18/M/2y	Cerebellar ataxia	normal	normal	-	CS	Recovered	Weihua Z et al (2022) [17]
19/M/4y	Cerebellar ataxia, seizures (including FBDS), encephalopathy	Background slowing	PET: significantly increased metabolism in bilateral basal ganglia and mildly increased in the medial temporal lobes	LEV, CZP	IVIG, CS	Recovered	Weihua Z et al (2022) [17]
20/F/14y	Movement disorders, ataxia, dysarthria, psycho-behavioral changes, hyponatremia,	normal	MRI: hyperintensities in the left frontal cortex, right medial temporal lobe, left insula and corpus striatum, right inferior frontal region, and left temporal lobe	LEV	CS, IVIG	Improved	Özçifçi G et al (2022) [18]
21/F/11y	Seizures	Background slowing and epileptiform discharge	normal	OXC	IVIG	Recovered	Wang Y et al (2023) [19]
22/M/4y	Seizures, psycho-behavioral changes	Background slowing and epileptiform discharge	MRI: mild atrophy on the cortex of left hemisphere	VPA, OXC, LCM	IVIG, CS, RTX, CLB	Improved	Wang Y et al (2023) [19]
23/M/17y	Seizures	Background slowing and epileptiform discharge	normal	VPA, OXC	IVIG, CS	Recovered	Wang Y et al (2023) [19]
24/F/6y	Seizures (including FBDS), movement disorders, ataxia, psycho-behavioral changes, amnesia	Focal slow and sharp waves	MRI: T2 and FLAIR high signal intensity in the left anterior putamen and right caudate nucleus	PB, OXC, VPA, LTG	CS, IVIG, RTX, TCZ	Improved	Jang S et al (2023) [20]
25/F/6y	Seizures (including FBDS), hyponatremia	Epileptiform discharge	MRI: an increased signal in the left temporal pole on T2FLAIR	PER, LCM	IVIG, CS, MMF	Recovered	This study

*Pt* patient, *M* male, *F* female, *y* year, *EEG* electroencephalogram, *MRI* magnetic resonance imaging, *PET* positron emission tomography, *LEV* levetiracetam, *CS* corticosteroid, *IVIG* intravenous immunoglobulin, *PE* plasma exchange, *MMF* mycophenolate mofetil, *ANS* automatic nervous system, *Aza* Azathioprine, *VPA* valproicacid, *LCM* lacosamide, *N/A* not available, *FBDS* faciobrachial dystonic seizures, *CBZ* carbamazepine, *TP* thiopental, *PB* phenobarbitone, *PHT* phenytoin, *TPM* topiramate, *RTX* rituximab, *CZP* clonazepam, *OXC* oxcarbazepine, *CLB* clonazepam, *TCZ* Tocilizumab, *LTG* lamotrigine, *PER* Perampanel

**Table 2:** Main data in the patients (including our patient) with anti-LGI1 encephalitis

Clinical features	LGI-positive encephalitis (*n*=25)
Seizures	16/25 (64%)
Encephalopathy	10/25 (40%)
Psycho-behavioral changes	12/25 (48%)
Sleep disorders	5/25 (20%)
Pain	4/25 (16%)
Amnesia	4/25 (16%)
Movement disorders	6/25 (24%)
FBDS	4/25 (16%)
Hyponatremia	3/25 (12%)
Ataxia	4/25 (16%)
ANS	2/25 (8%)
Dizzy	2/25 (8%)
Abnormal brain MRI/PET	11/25 (44%)
Abnormal EEG	15/25 (60%)
Immune therapy	20/25 (80%)

*LGI1* Leucine-rich glioma-inactivated protein 1, *FBDS* faciobrachial dystonic seizures, *ANS* automatic nervous system, *EEG* electroencephalogram, *MRI* magnetic resonance imaging, *PET* positron emission tomography

Brain MRIs or positron emission tomographies (PETs) were available for 24 patients (one was not given) [5] and revealed abnormalities in 11 patients [5, 9–12, 17–20]: brainstem hyperintensities (1 case) [5], mild atrophy on the cortex of left hemisphere (1 case) [19], bilateral basal ganglia (2 cases) [17, 20], limbic lobe (temporal lobe or hippocampus) hyperintensities (7 cases) [5, 9–12, 18].

Of the 23 patients for whom response and outcome data were available, 20 patients received immunotherapy (intravenous immunoglobulin, corticosteroid, plasmapheresis, azathioprine, mycophenolate mofetil, rituximab, tocilizumab) [5, 7–20], while 3 patients received only symptomatic therapy [5]. Both types of therapy were found to be beneficial, resulting in full recovery for 11 patients [7, 9, 12, 14, 16, 17, 19]. One patient initially responded well to first-line immunotherapy with corticosteroids, IVIG but suffered a relapse after 7 months. He did not fully recover until he underwent treatment with rituximab [15].

## Discussion and conclusions

According to the Chinese expert consensus on the diagnosis of autoimmune encephalitis, anti-LGI1 encephalitis can be diagnosed using the following criteria: acute or subacute onset with progressive aggravation; clinical signs of limbic encephalitis or FBDS; normal leukocyte count or mild lymphocyte reaction on CSF examination; abnormal brain MRI signals in the bilateral or unilateral medial temporal lobe; abnormal EEG activity; and serum and/or CSF anti-LGIl antibody positivity. Our case presented with an acute onset of seizure, a normal leukocyte count on CSF examination, and abnormal brain MRI signals in the unilateral temporal lobe. These symptoms, combined with the abnormal EEG findings and positive anti-LGIl antibody in the serum and CSF, supported the diagnosis of anti-LGI1 encephalitis.

LGI1 is a secreted neuronal protein that interacts with the presynaptic protein, a disintegrin and metalloproteinase 23 (ADAM23); and the postsynaptic protein, a disintegrin and metalloproteinase 22 (ADAM22). These interactions lead to the organization of a trans-synaptic protein complex that involves pre-synaptic Kv1.1/Kv1.2 potassium channels and post-synaptic AMPAR scaffolds [21].

LGI1 is mainly present in the hippocampus and temporal cortex [22]. According to previous reports, in LGI1 IgG-positive cases, most patients’ brain MRI or PET showed lesions in mesial temporal lobe [23]. In this pediatric cohort, about 44% of patients revealed abnormalities in Brain MRIs or PETs, with lesions primarily in the temporal lobe or hippocampus, which is similar to the adult patients. In our case, the patient’s brain MRI showed an abnormality in the left temporal pole, leading to suspicion of limbic encephalitis. An antineuronal antibody test was performed on both serum and CSF. All patients had LGI1-IgG in their serum or/and CSF in this pediatric cohort.

Importantly, pediatric cases appear to show distinctly different clinical presentations compared to adult patients. For example, men were more commonly affected than women (66% were men) [24]. In contrast, among the pediatric patients described, we observed a greater prevalence of females (60%) than males (40%) in pediatric patients. In adult patients, FBDS and hyponatremia were typically identified as hallmark symptoms [25], but only 4 patients had FBDS [15, 17, 20] and 3 patients had hyponatremia in our cohort [15, 18]. Frequent seizures, encephalopathy, psycho-behavioral changes were the most common neurological accompaniments of anti-LGI1 encephalitis in our pediatric patients. Indeed, focal seizures were found to be more common than FBDS. However, at times, it can be challenging to differentiate between FBDS and focal-onset seizures. Three patients with hyponatremia were reported [15, 18], which differed from the patients described in the original articles [5]. The absence of apparent cancer was consistent with previously reported cases of pediatric patients [5, 26]. In addition, isolated symptoms may be observed in some pediatric patients with anti-LGI1 encephalitis, which can make it challenging to identify certain clinical presentations such as neuropathic pain or memory disorders, particularly in very young patients. Diagnosing anti-LGI1 encephalitis early based on clinical features alone can be challenging in young patients.

The therapeutic strategies used in pediatric patients generally led to positive outcomes [5, 7–20]. Three patients received only symptomatic therapy [5]. According to previous reports, the natural history of anti-LGI1 encephalitis was variable, and spontaneous complete recovery may occur without immune therapy in adult patients [26]. This pattern has also been observed in pediatric patients [5]. Nosadini et al. [26] also found no correlation between time to immune therapy and positive outcomes, and patients who received second-line immune therapy had a higher relapse rate than patients treated with first-line immune therapy only in adult patients. However, this has not been reported in pediatric patients and needs to be confirmed by more cases.

In conclusion, the characteristics of anti-LGI1 encephalitis in children differs from those in adults. However, the reports about pediatric LGI1-antibody encephalitis are rare. It is crucial to collect and analyze data from more cases to promote accurate diagnosis and timely treatment.

## Data Availability

The raw datasets generated and analyzed during the current study are not publicly available in order to protect participant confidentiality. The datasets obtained during the current study are available from the corresponding author if the requirements are reasonable.

## Abbreviations

LGI1: Leucine-rich glioma-inactivated protein 1
FBDS: Faciobrachial dystonic seizures
MRI: Magnetic resonance imaging
VGKC: Voltage gated potassium channel
CASPR2: Contactin-associated protein like-2
VEEG: Electroencephalogram
CSF: Cerebrospinal fluid
CT: Computed Tomography
MMF: Mycophenolate mofetil
PET: Positron Emission Tomography
IVIG: Intravenous immunoglobulin
ADAM23: A disintegrin and metalloproteinase 23
ADAM22: A disintegrin and metalloproteinase 22
AMPA: α-amino-3-hydroxy-5- methyl-4-isoxazolepropionic acid
